# COVID-19-Associated Acute Transverse Myelitis: A Case Series of a Rare Neurologic Condition

**DOI:** 10.7759/cureus.18551

**Published:** 2021-10-06

**Authors:** Liaquat Ali, Imran Mohammed, Yasin Zada, Haya Salem, Ambreen Iqrar

**Affiliations:** 1 Neurology, Hamad General Hospital, Doha, QAT; 2 Neurology, Weill Cornell Medicine-Qatar, Doha, QAT; 3 Internal Medicine, Hamad General Hospital, Doha, QAT; 4 Medicine, College of Medicine, Qatar University, Doha, QAT

**Keywords:** interleukin-6 (il-6)., blood-brain barrier (bbb), angiotensin-converting enzyme 2 (ace-2), cerebrospinal fluid (csf), acute transverse myelitis (atm)

## Abstract

Severe acute respiratory syndrome coronavirus 2 (SARS-CoV-2) viral infection is not confined to the respiratory system, but has also shown extra-pulmonary invasion including the nervous system. About 36.4% of hospitalized patients in China with confirmed coronavirus disease 2019 (COVID-19) infection had neurological manifestations. SARS-CoV-2 virus enters the human body through angiotensin converting enzyme-2 (ACE-2) receptors on the surface of human cells and causes disease. ACE2 receptors are also expressed on the surface of spinal cord cells. More rare neurologic conditions have been reported in the literature to be associated with COVID-19 such as acute transverse myelitis (ATM), Guillain Barre syndrome, acute flaccid myelitis, etc. We report two cases of confirmed COVID-19 who presented four to five days of their COVID-19 symptoms and progressive bilateral lower limb weakness and urinary retention.

ATM is an acquired spinal cord disorder. ATM is a relatively common neurological complication of COVID-19, accounting for 1.2% of all neurological complications associated with COVID-19. The mechanism by which COVID-19 causes ATM is not completely understood but has been assumed to be due to the structural resemblance of RNA viruses. Entrance of SARS-CoV-2 to the nervous system can take place through two pathways, either directly or indirectly. The direct pathway is through trans-synaptic transmission from the peripheral nervous system or by hematogenous spread into the blood-brain barrier through ACE-2, while the indirect pathway is through a systemic immune response.

## Introduction

Since the revelation of coronavirus disease 2019 (COVID-19) globally from Wuhan, China, the infection has progressively evolved in presentation. We now know that the viral infection is not confined to the respiratory system, through which it is transmitted, but has also shown extra-pulmonary invasion including the nervous system at different stages of the infection course. The most common neurological symptoms in COVID-19 patients are anosmia, myalgia, headache, altered mental status or brain fog. About 36.4% of hospitalized patients in China with confirmed COVID-19 infection had neurological manifestations [1]. The same study also revealed that patients with a more severe infection were more likely to develop neurological manifestations. The severe acute respiratory syndrome coronavirus 2 (SARS-CoV-2) virus enters the human body through angiotensin converting enzyme-2 (ACE-2) receptors on the surface of human cells and causes disease [2,3]. ACE2 receptors are also expressed on the surface of spinal cord cells; whether spinal cord neurons are concerned in COVID-19 is still unknown [4,5]. A spectrum of neuroimaging abnormalities has been described in patients with COVID-19, the most common of which are acute ischemic stroke, cortical FLAIR signal abnormality, cerebral microbleeds, leptomeningeal enhancement, cytotoxic lesions in the splenium of the corpus callosum and other manifestations of encephalitis [6].

More rare neurologic conditions have also been reported in the literature to be associated with COVID-19 such as cerebrovascular accidents, acute transverse myelitis (ATM), Guillain Barre syndrome (GBS), acute flaccid myelitis, acute encephalitis, acute disseminated encephalomyelitis (ADEM), generalized myoclonus and posterior reversible encephalopathy syndrome (PRES) [7]. In this case series, we present peculiar neurological complications of COVID-19-related ATM in patients who were admitted to the COVID-19 facility, Tertiary Care Hospital, Hamad Medical Cooperation in Doha, Qatar.

## Case presentation

Case 1

**Figure 1:**
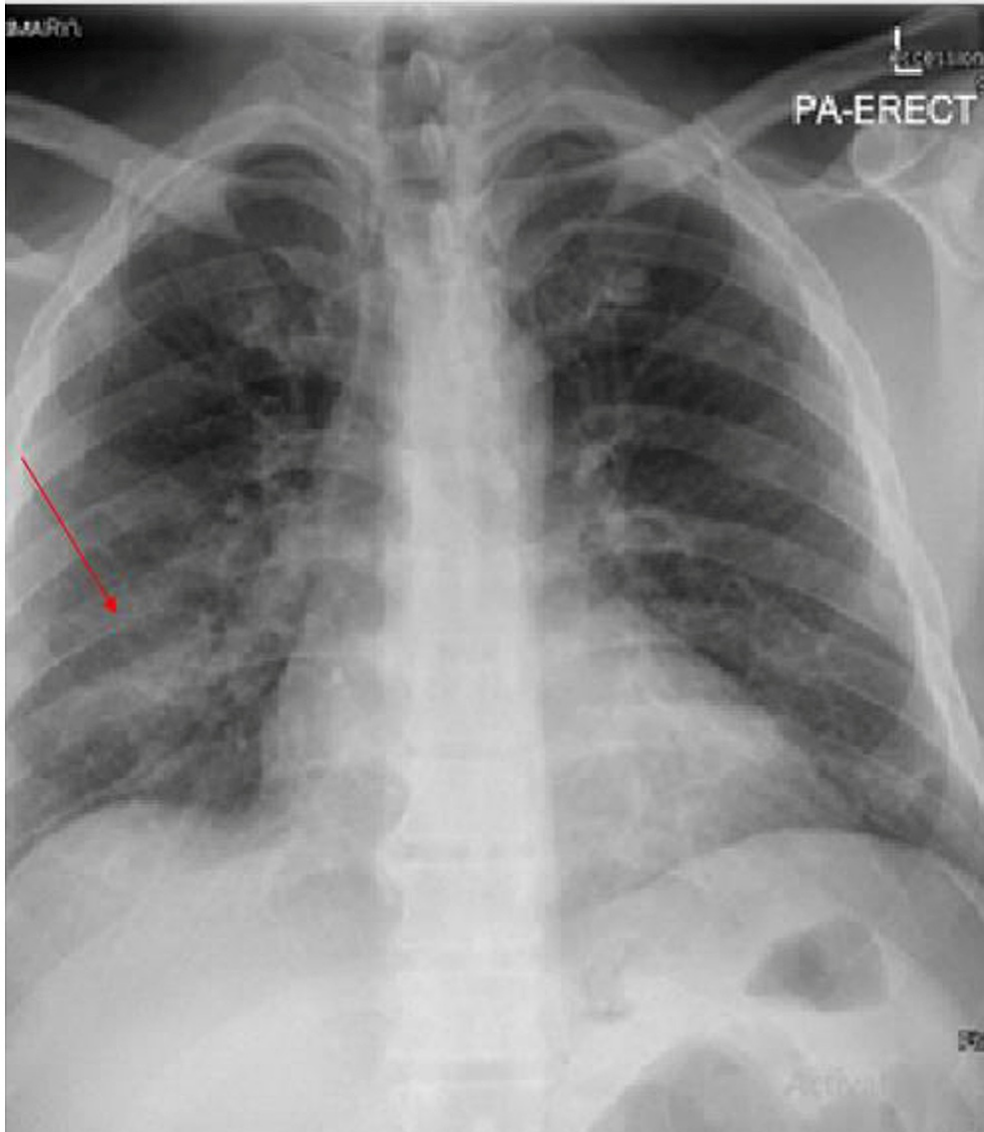
Chest x- ray (PA view) Chest x-ray showed right side lower zone evolving consolidation, extending to periphery and perihilar regions.

A 56-year-old gentleman, known to have type 2 diabetes mellitus for four years and newly discovered G6PD deficiency, came to the emergency department (ED) with complaints of high-grade fever, fatigue for four days, lower limbs weakness and urinary retention for one day. He reported no contact with COVID-19-positive or other sick patients. On presentation to the emergency department, he was febrile with a temperature of 38.9°C, normal respiratory rate and oxygen saturation were 96% at room air. Physical examination revealed lower abdominal tenderness, palpable urinary bladder, bilateral lower limbs weakness of 3/5 medical research council (MRC) grades for muscle strength and numbness. Foley’s catheter was inserted which drained more than a liter of urine in the urinary bag. Ultrasound kidney, ureter and bladder (KUB) was performed to rule out obstructive uropathy that showed distended urinary bladder, pre-void urine volume of 1327ml, post-void 873ml. Chest X-ray showed evolving consolidation in the right lower zone and perihilar extending to the periphery and bilateral prominent broncho-vascular marking (Figure 1).

**Figure 2:**
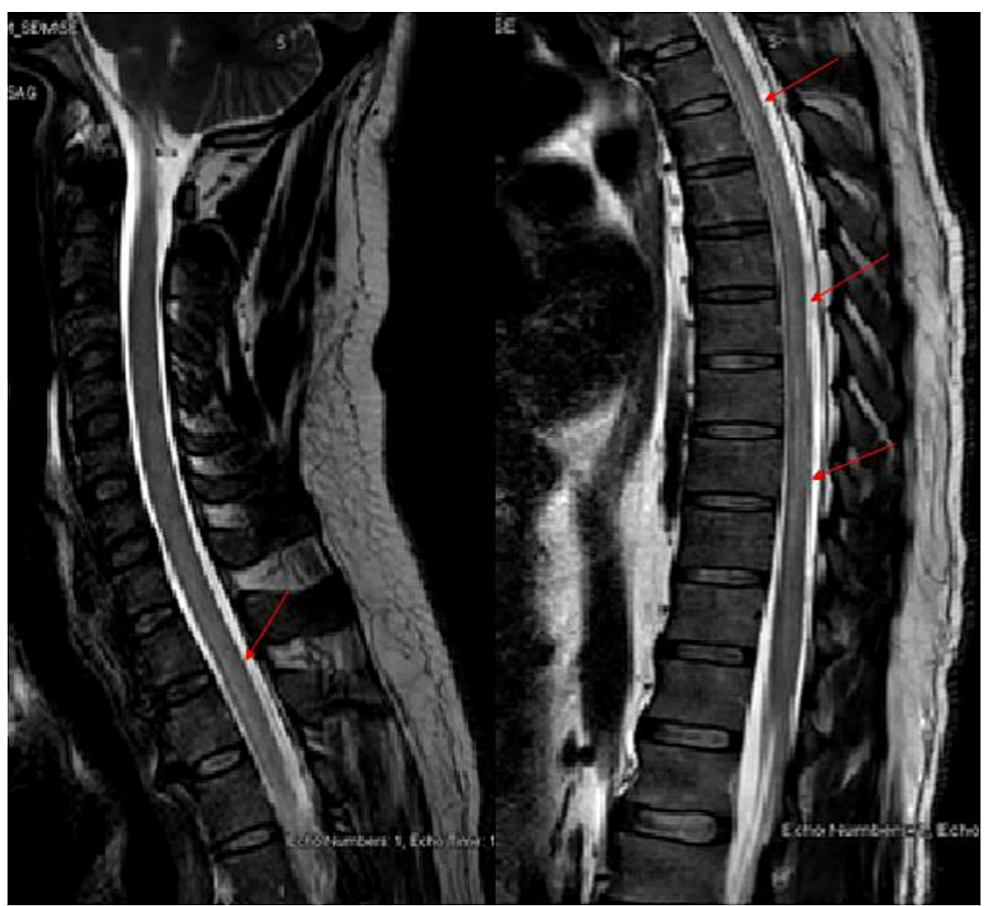
MRI spine with gadolinium Magnetic resonance imaging (MRI) with gadolinium T2 weighted images of the cervicothoracic spine showed subtle high signal intensity in the ventral horn of the grey matter in the upper and mid thoracic cord, without post IV contrast enhancement (red arrow head).

**Figure 3:**
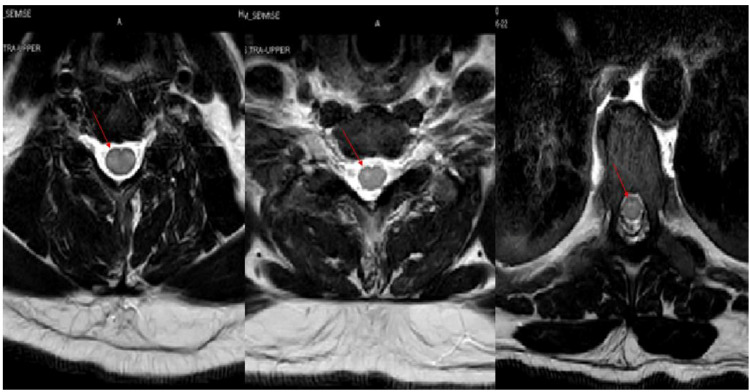
MRI cervicothoracic spine with gadolinium. MRI cervicothoracic spine T2 weighted images of the axial view with gadolinium showed hyperintensity signals in the ventral horn of the grey matter in the upper and mid thoracic cord without enhancement (red arrow head).

The patient was admitted to the medical floor for evaluation of fever and suspected COVID-19. After 12 hours of admission, the patient became tachypneic and hypoxic with a respiratory rate of 36 breaths/minute and pulse oximetry oxygen saturation drop to 92% at room air. COVID-19 PCR sample obtained from both nasopharynx and throat of the patient was positive for SARS-CoV-2 infection. He was diagnosed with COVID-19 pneumonia. He was transferred to COVID-19 tertiary care hospital for further evaluation and treatment. The neurology team was consulted for evaluation of lower limbs weakness. Neurological examination showed normal higher mental function. All cranial nerves included facial nerve and extraocular muscles movements were intact and full range. Motor system examination of upper limbs showed good muscle bulk, normal tone and muscles strength, while the lower limb examination revealed reduced muscles tone, muscles strength of 3/5 (MRC) distal and proximal bilaterally. Reflexes were no response (deep tendon reflexes grade of 0) at knee and ankle reflexes and equivocal plantar response. There were impairment of sensation of lower limbs bilateral with a sensory level at T6 (at xiphoid process). MRI Head and Spine with gadolinium of T2 weighted images of the sagittal and axial spine demonstrate subtle high signal intensity in the ventral horn of the grey matter in the upper and mid thoracic cord, without post gadolinium contrast enhancement; findings represent viral transverse myelitis related to COVID-19 infection (as shown by red arrows in Figure 2 [sagittal view] and Figure 3 [axial view]).

Lumbar puncture was performed which showed cerebrospinal fluid (CSF) pleocytosis with lymphocyte predominant, normal glucose and high protein, Mycobacterium tuberculosis (MTB) PCR and herpes simplex virus (HSV) PCR were negative (Table 1). Other laboratory results that were performed during admission are tabulated in Table 2 including negative autoimmune immunological tests.

**Table 1:** Cerebrospinal fluid analysis and cultures Lumbar puncture was performed showed of cerebrospinal fluid (CSF) pleocytosis with lymphocyte predominant, normal glucose and high protein. MTB: Mycobacterium tuberculosis, HSV: herpes simplex virus

CSF analysis	Results	Reference range
Appearance	Clear	
White Cell count	152 (89% lymphocytes)	(0-5 /uL)
Protein	0.54 gm/L	(0.15 – 0.45 gm/L)
Albumin	400 (high)	(0-350mg/L)
Glucose	4.69 mmol/L	(2.22 – 3.89 mmol/L)
Viral meningitis PCR panel (include HSV1,2)	Negative	
Gram stain and culture	Negative	
CSF Cryptococcal antigen	Negative	
CSF gram stain/cultures	No growth	
CSF MTB -PCR	Negative	

**Table 2:** Routine blood Investigations. Blood lab Investigations done at the Emergency Department. INR: international normalized ratio, ALT: alanine transaminase, AST: aspartate transaminase, HbA1c: hemoglobin A1c, HSV: herpes simplex virus, ANA: antinuclear antibodies, ANCA: antineutrophil cytoplasmic antibodies, TSH: thyroid stimulating hormone

Test	Result (Reference range)	
White blood cell	10.2 (3.5-9.5×10^9^ /L)	
Hemoglobin	10.6 (13.5-17.5g/dL)	
INR	1.1 (<1.1)	
creatinine	61 (44-110umol/L)	
Prothrombin time	14 (11 to 13.5 sec)	
Sodium	135 (135-149 meq/L)	
ALT	62 (5-40 U/L）	
AST	125 (5-40 U/L）	
C-reactive protein	78 (0-5mg/L)	
Ferritin	435 (30-553 ug/L)	
Interleukin-6 (IL-6)	97 (high) (≤ 7 pg/mL)	
D-Dimer	23.5 (0.0- 0.49 mg/L FEU)	
HbA1c	5.7%	
S HSV IgG/M	Negative	
ANA	Negative	
ANCA	Negative	
TSH	0.47(normal)	
COVID-19 PCR	Positive	
Vit B12 level	109 low (145-595pmol/L)	
Troponin –T hs	63 high (3-15 ng/L)	
Blood culture	No growth	

After 48 hours of admission, the patient’s inflammatory markers were noted to be elevated with high c-reactive protein (CRP), D-dimer, ferritin, interleukin-6, troponin T, alanine transaminase (ALT) and aspartate transaminase (AST) as shown in Table 2. Based on the presenting symptoms, physical examination, MRI of spine and laboratory results of acute weakness of lower limbs, urinary retention, an abnormal sensory level at T6, abnormal CSF study and abnormal MRI of the spinal cord which demonstrated high signal intensity in the upper and mid-thoracic cord, findings were suggestive of the diagnosis of acute transverse myelitis, most likely related to COVID-19. The patient was treated with intravenous pulse methylprednisolone 1 gram daily for five days and acyclovir 10 mg/kg three times for 14 days. On the fourth day of admission in the COVID-19 facility, the patient collapsed and went into cardiac arrest. Immediate cardiopulmonary resuscitation was initiated. After a few cycles of resuscitation return of spontaneous circulation was achieved but the patient remained unconscious. Bedside “point of care” ultrasound exam (POCUS) showed right atrial and ventricle (RA/RV) dilatation. A massive unstable pulmonary embolism was suspected as the cause of cardiac arrest and he was treated with thrombolytic therapy. Despite this the patient deteriorated and unfortunately passed away.

Case 2

**Figure 4:**
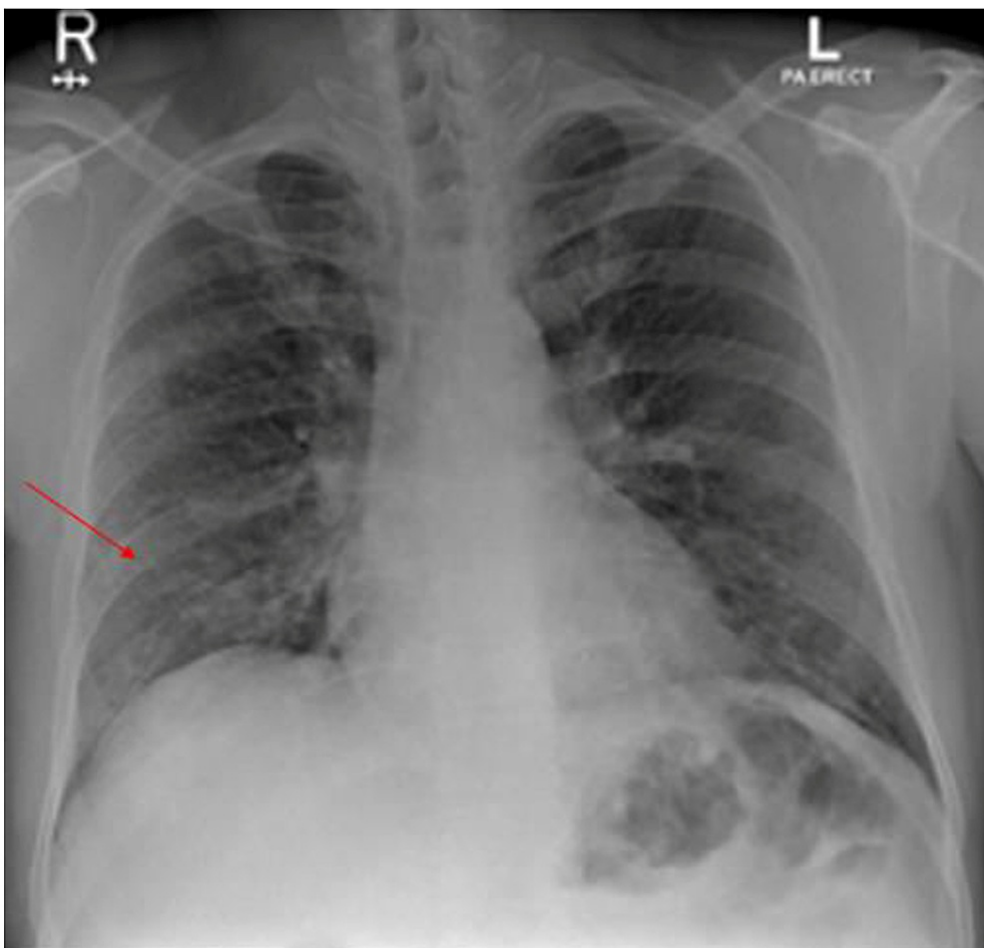
Chest X ray (case 2) Chest X-ray showed multiple airspace heterogenous opacities in both lung fields especially lower and peripheral lung zones (red arrow head).

A 43-year-old gentleman, newly diagnosed with diabetes mellitus and not previously vaccinated for COVID-19, was referred to the Emergency Department (ED) with a five-day history of fever, sore throat, headache, and generalized body ache. He denied any history of sick contacts. He was found to be positive for COVID-19 reverse transcription polymerase chain reaction (RT-PCR) test from nasopharyngeal and oropharyngeal swabs. Upon arrival at the ED, his chest x-ray showed multiple airspace heterogenous opacities in both lower and peripheral lung fields (Figure 4).

His highest body temperature recorded in the ED was 39.4 F. Two hours after admission, the patient complained of sudden onset bilateral lower limb numbness and weakness, inability to sit, and difficulty passing urine with intermittent urinary dribbling. The weakness was isolated to his lower limbs and was symmetrical. He had no history of spinal trauma.

**Figure 5:**
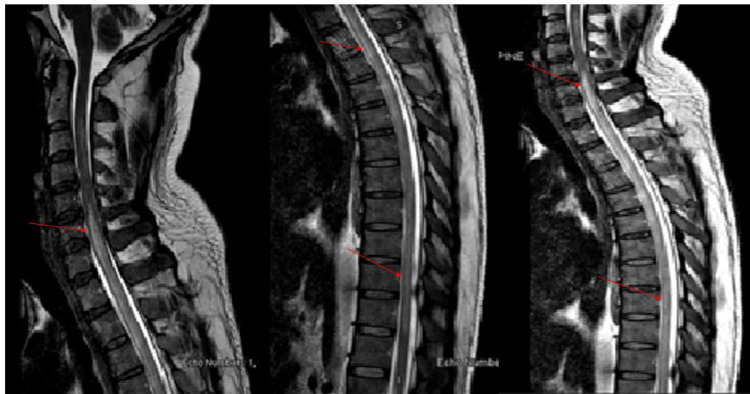
MRI cervicothoracic spine with gadolinium. MRI of the cervicothoracic spine T2 weighted images sagittal view showed extensive intramedullary abnormal cord signaling extending from cervical C-2 to thoracic T-11 vertebral level without post contrast enhancement.

**Figure 6:**
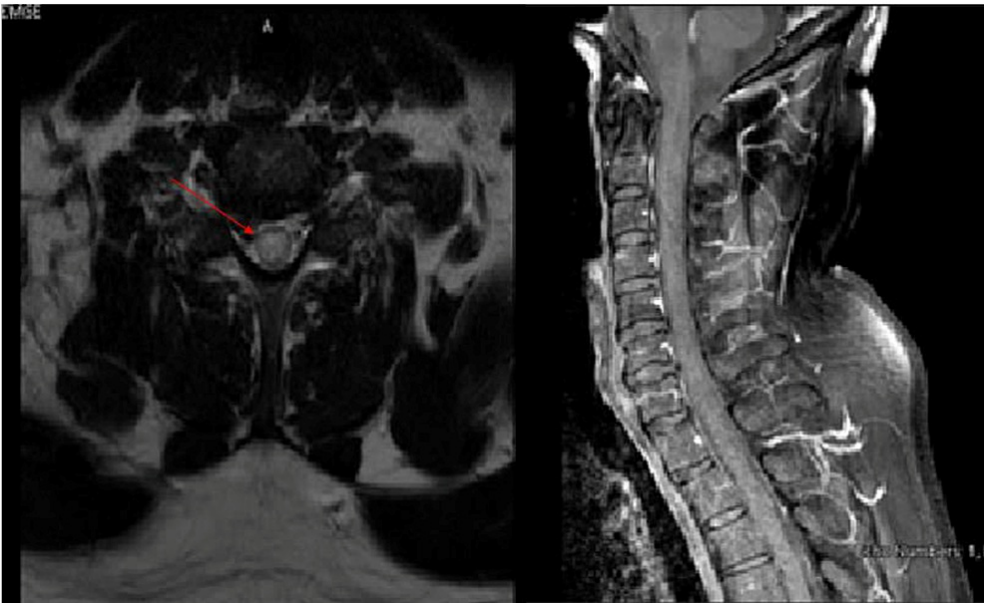
MRI cervicothoracic spine with gadolinium. MRI of the spine cervicothoracic spine T2 weighted axial view showed intramedullary extensive abnormal cord signaling extending from cervical C2 to thoracic T11 vertebral level (as shown in 6A), and T1 weighted sagittal view with post gadolinium without post contrast enhancement (as shown in 6B).

On physical examination, the patient vital signs were stable; oxygen saturation was 98% on room air. Neurological examination revealed intact higher function, cranial nerve and upper limbs tests. Meanwhile, on examination of the lower limbs, the patient was paraplegia, MRC muscle power of 0/5 that is symmetric proximally and distally - with pronounced hypotonia bilaterally along with symmetrical loss of pinprick sensation below the level of T10, proprioception hypesthesia, and hyporeflexia at the knee and ankle. Additionally, the patient had suprapubic tenderness with a palpable bladder and loss of anal tone. Insertion of foley catheters in the ED yielded more than 600 ml of urine. Magnetic resonance imaging (MRI) of the head and spine were ordered. MRI of the head with gadolinium was unremarkable and ruled out brain structural or demyelinating disease, while MRI of the spine with gadolinium showed longitudinally extensive abnormal cord signaling extending from cervical C-2 to thoracic T-11 vertebral level without enhancement or haemorrhage or no diffusion restriction and normal appearance of conus medullaris and cauda equina nerve roots (Figure 5, Figure 6).

Cerebrospinal fluid (CSF) analysis showed pleocytosis with predominantly neutrophils with elevated protein and glucose levels and negative oligoclonal bands. Bacterial and tuberculosis cultures showed no growth and viral PCR meningitis panel, MTB-PCR of the CSF was negative (Table 3).

**Table 3:** Cerebrospinal fluid analysis and cultures. Cerebrospinal fluid (CSF) analysis showed predominantly neutrophilic pleocytosis with elevated CSF protein and glucose and negative oligoclonal bands, bacterial antigens and cultures showed no growth and viral PCR meningitis panel of the CSF was negative including HSV 1, 2. MTB: Mycobacterium tuberculosis, HSV: herpes simplex virus

CSF analysis	Results	Reference range
Appearance	Clear	
White Cell count	186 (85% neutrophils)	(0-5 /uL)
Protein	1.25 gm/L	(0.15 – 0.45 gm/L)
Albumin	715 (high)	(0-350mg/L)
Glucose	9.16 mmol/L	(2.22 – 3.89 mmol/L)
Viral meningitis PCR panel (include HSV1,2)	Negative	
Gram stain and culture	Negative	
Oligoclonal bands	Negative	
CSF-IgG	208(high)	(0-34mg/L)
Cryptococcal antigen	Negative	
IgG index	0.6	(0.3-to-0.6)
CSF MTB -PCR	Negative	

Other laboratory results that were performed during admission are tabulated in Table 4, including negative autoimmune immunological tests.

**Table 4:** Blood lab Investigations. Other blood laboratory results that were performed during admission, including negative autoimmune immunological tests. INR: international normalized ratio, HbA1c: hemoglobin A1c, ANA: antinuclear antibodies, ANCA: antineutrophil cytoplasmic antibodies

Test	Result	Reference range
White blood cell	10.8 with anc of 8%	(3.5 to 9.5 x 10^9 ^/L)
Hemoglobin	14.8	(13.5 to 17.5 gm/dl)
INR	1.2	(0.8 to 1.1))
Urea	3.3	(2.1 to 8.5 mmol/l)
Prothrombin time	14.3	(11 to 13.5 seconds)
Sodium	135	(135 to 149meq/l)
C-reactive protein	36.1	(8 to 10 mg/l)
Ferritin	338	(24 to 336 mg/l)
HbA1c	11.4%	(4 to 5.6%)
Random blood sugar	15.3 mmol/L	(7.8 to 11 mmol/l)
ANA	Negative	
ANCA	Negative	
Anti-Ro/La	Negative	
COVID-19 PCR	Positive (CT=24.38)	
Vit B12 level	291	(145-595pg/ml)

Based on clinical and radiological findings the patient was diagnosed with para-infectious long extensive transverse myelitis secondary to symptomatic COVID-19 infection.

**Figure 7:**
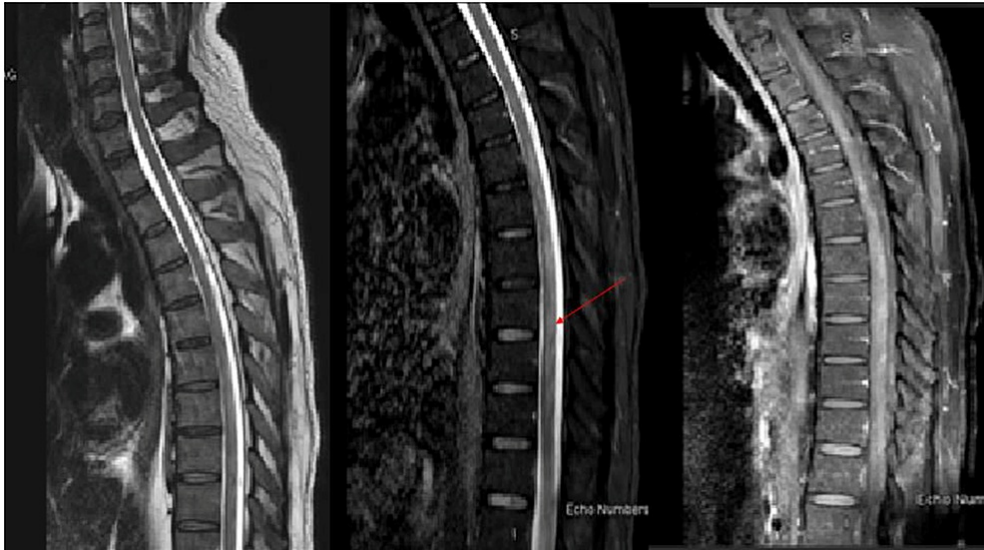
MRI cervicothoracic spine with gadolinium MRI of the T2 weighted cervicothoracic spine was repeated after two weeks showed significant interval regression of the spinal cord extensive and abnormal signal intensity seen in the cervical and thoracic cord, with minimal residual abnormal signal intensity seen in the mid dorsal region from T5 to T9.

The patient was treated with intravenous pulse methylprednisolone 1 gm daily for five days. Adjacently, the patient received five days of IV immunoglobulin (0.4g/kg) and extensive physiotherapy. Post-treatment, the patient did not show any clinical improvement, therefore, MRI of the spine was repeated which showed substantial interval regression in radiological findings with with minimal residual abnormal signal intensity seen in the mid dorsal region from T5 to T9. No definite diffusion restriction or focal enhancing lesion (Figure 7).

As a result, the team decided to begin five sessions of plasmapheresis and refer the patient for rehabilitation therapy afterwards. During his admission, there was no improvement in the patient's paraplegia. He was transferred to a rehabilitation center for intensive physiotherapy where he stayed for two and half months. At the time of discharge examination from the rehabilitation center, although there was an improvement in overall functional status like sitting balance, independent bed mobility, improvement in lower limb spasticity but the sensations in lower limbs remained absent with power in lower limbs graded as 0/5 and neurogenic bladder. He was dependent on a self-propelled wheelchair for mobility. At this point, the patient was discharged to his country with advice to continue the rehabilitation program.

## Discussion

Acute transverse myelitis (ATM) is an acquired spinal cord disorder that presents with sudden-onset of varying degrees of motor weakness, sensory loss, and bowel and/or bladder dysfunction due to an immunological reaction, that could be supported by MRI findings [8]. The incidence of acute transverse myelitis is up to 3 per 100,000 patient years (0.003%) [9]. The etiology of ATM can vary from inflammatory, demyelinating, autoimmune, infectious, toxin-induced and paraneoplastic factors. According to a review article published in April 2021, ATM has proved to be a relatively common neurological complication of COVID-19, accounting for 1.2% of all COVID-19 neurological complications [10]. The Oxford-AstraZeneca COVID-19 vaccine consists of a replication-deficient chimpanzee adenoviral (ChAdOx1 ) containing SARS-CoV-2 structural surface vector glycoprotein antigen (spike protein) gene. Three vaccine-related ATM cases have been reported due to the Oxford-AstraZeneca COVID-19 vaccine [11,12].

The dormancy period between the time of infection with COVID-19 and developing ATM is unknown, especially since some patients have an asymptomatic infection course. In our cases, there were antecedent respiratory and systemic symptoms for almost four to five days while in the first reported case of ATM due to COVID-19 is a 66-year-old patient in China, neurological features presented on day 10 of respiratory symptoms [10]. 

The mechanism by which COVID-19 causes ATM is not completely understood but has been assumed to be similar to that of severe acute respiratory syndrome coronavirus 1 (SARS-CoV-1) that arose in 2002 due to the structural resemblance of both RNA viruses. SARS-CoV-1 was thought to cause extra-pulmonary manifestations through its functional receptor, ACE-2, which is abundantly expressed on the endothelial layer of blood vessels of all organs. Entrance to the nervous system can take place through two pathways: either directly or indirectly. The direct pathway is through trans-synaptic transmission from the peripheral nervous system since neurons do not express the ACE-2 receptor themselves or by hematogenous spread into the blood-brain barrier (BBB) through ACE-2 [13]. On the other hand, the indirect pathway is through a systemic immune response that prompts the release of a cytokine storm, especially interleukin-6 (IL-6). Consequently, these proinflammatory cytokines increase the vascular permeability of the BBB and enhance the dissemination of the virus. Further, an exaggerated autoimmune cytokine response will likely induce incessant multiorgan failure and eventually the death of the patient [14]. This autoimmune theory has been also sustained by the positive patient response to steroids in one case report from Dubai [15]. However, in one of our cases, and two other reported cases in China and India, patients did not show satisfactory improvement in symptoms after receiving steroids, despite observing radiological amelioration in one of our patients particularly. Hence, consensus on whether the mechanism is direct viral neurotropism or an autoimmune has not been attained yet. In favor of the autoimmune theory, more research is required to explore immunomodulating treatments that allow for a balance between a forceful inflammatory response that is beneficial to encase the viral infection and an unexaggerated cytokine response to avoid multiorgan involvement. Even though, interestingly, our patient’s baseline symptoms and lab: results did not deteriorate after receiving several subsequent immune-modulating treatment modalities.

In the group of hospitalized patients with COVID-19 from three centers in China, it was reported that the development of neurological complications was significantly more in those patients with a severe infection status, occurring in 45.5% of them [1]. The severity of COVID-19 pneumonia was determined in concordance with the criteria set by the American Thoracic Society. Nonetheless, our patient was classified as having a non-severe infectious course as per the guidelines, yet developed ATM. In another study, Sabaka et al. suggested the use of IL-6 to predict the severity and need of hospitalization of COVID-19 confirmed patients due to its representation of the inflammatory state; however, we did not measure levels of IL-6 in our second case while in the first case it was high [16]. 

Another possible contributing factor to developing complications secondary to COVID-19 could be attributed to the patient’s vaccination status, since Thompson et al. found that the mean viral load in patients who were partially or full vaccinated was 40% less than those who were unvaccinated at all, like our patient [17]. 

## Conclusions

This case series represents patients with COVID-19-associated acute transverse myelitis, a rare neurologic complication that was supported by abnormal MRI spinal cord and CSF findings. The exact mechanism of COVID-19-associated ATM is multifactorial and may arise from direct effects of the virus as well as a systemic response to SARS-CoV-2 infection. These cases show that we need to be vigilant of the neurological complications of COVID-19 infections and help physicians in the early diagnosis of rare COVID-19-associated acute transverse myelitis.
